# Prevalence and causes of visual impairment among Saudi adults

**DOI:** 10.12669/pjms.331.11871

**Published:** 2017

**Authors:** Mujeeb Ur Rehman Parrey, Farhan Khashim Alswelmi

**Affiliations:** 1 Dr. Mujeeb Ur Rehman Parrey, Ph.D. Faculty of Medicine, Northern Border University, Arar, Kingdom of Saudi Arabia; 2 Dr. Farhan Khashim Alswelmi, M.D. Faculty of Medicine, Northern Border University, Arar, Kingdom of Saudi Arabia

**Keywords:** Visual impairment, Blindness, Refractive error, Cataract, Diabetic retinopathy

## Abstract

**Objective:**

To evaluate the prevalence and causes of visual impairment (VI) among Saudi adults in Arar City, the capital of Northern Border Region of Saudi Arabia.

**Methods:**

This population-based cross-sectional study was conducted on 705 Saudi adults aged 18 years and older. All participants underwent ophthalmic examination including visual acuity (VA) testing, auto refraction, intraocular pressure (IOP) measurement and fundus examination.

**Results:**

In the present study 166 cases (23.5%) were found to have VI, while only 12 cases (1.7%) were considered as blind following the WHO definitions. Cataract was found to be the main cause of VI [51 cases (30.7%)] followed by refractive error (RE) [41 cases (24.7%)] and diabetic retinopathy (DR) [22 cases (13.2%)]. Seventy one cases of the studied subjects (10.07 %) had shown unilateral VI, while 13 cases (1.8%) had shown unilateral blindness with the other eye normal (VA ≥ 20/20).

**Conclusion:**

Visual impairment is highly prevalent among Saudi adults in Arar city. Cataract, refractive error and diabetic retinopathy are the main 3 leading causes. Better plans for diagnosis and treatment should be considered to decrease the magnitude of the problem.

## INTRODUCTION

The term “visual impairment”(VI) refers to a functional limitation of the eye (s) or visual system due to a disorder or disease that can reduces a person’s ability to perform his activities of daily life. According to the latest estimates, globally about 314 million people are visually impaired, of whom 45 million are blind.1 The WHO has estimated that almost 90% of the world’s visually impaired people live in low-income settings and over 80% of global VI is preventable or treatable.2 But, due to the lack of eye-care services, millions of people remain at risk of visual loss. To date, epidemiological surveys have been conducted in many countries worldwide.3-8

Only few studies of such nature have been carried out in Saudi Arabia which showed that the causes of VI vary in different age groups and in different geographical locations of the country.9-12 In the present study ophthalmic evaluation was conducted for adult population of Arar city to investigate the prevalence and highlight the main causes of VI in this part of the Kingdom and compare it with the data obtained from the other localities of the country and with the data concerning VI worldwide.

## METHODS

This study was conducted from January 2014 to June 2015. Free informed consent was obtained from all 705 subjects. Ethical approval (No. 1-001-435) was obtained from Ethics Committee of the Northern Border University. Study design: Saudi people aged ≥18 years were enrolled in the current study. The participants were screened in the free eye camps held at different locations in Arar and followed up at Arar Central Hospital. Each eye was tested separately unaided on Auto Chart Projector (TOPCON ACP-8 R). Persons wearing glasses were re-checked while wearing their own glasses. Autorefraction was performed on auto refractometer (Topcon KR 8900) on all subjects with VA below 20/30 in one or both eyes. Subjective refraction tests were performed to achieve the best corrected visual acuity (BCVA). For complete evaluation, slit lamp examination, direct and indirect ophthalmoscopy, IOP measurements were performed for all participants, while perimetry and corneal topography were carried out if required.

We used the World Health Organization (WHO) definition, in which visual impairment (VI) was categorized into mild VI for visual acuity (VA) ≥20/30 to ≤20/60, low vision [including moderate VI for VA ≥ 20/60 to ≤20/160 and severe VI for VA≥ 20/160 to ≤20/400] and blindness for VA <20/400 in the better seeing eye with the best correction.

Statistical analysis: Prevalence was calculated by dividing the number of visually impaired cases by the total number of the studied population, while their 95% confidence intervals (CI) were calculated following Newcombe.13 Chi-square test was used to get the nominal association. The analysis was done using Prism5 (GraphPad Software Inc., San Diego, CA). Significance was estimated with p-values<0.05.

## RESULTS

Demographic data: From the total surveyed 705 Saudi adults, 341 subjects were males (48.3%) and 364 females (51.6%). Their ages ranged from 18 -87 years with mean (42.5years±17.9years).

**Fig. 1:**
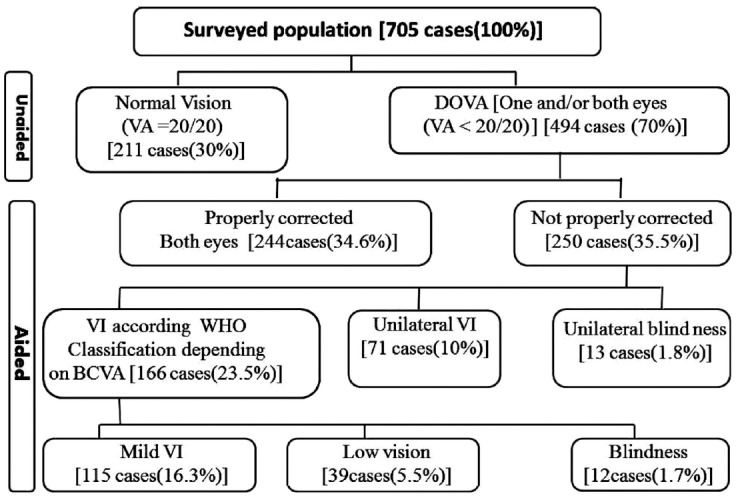
Outcome of the ophthalmic evaluation of the surveyed population.

Visual Acuity: VA data of the survived population are shown in Fig.1.

Prevalence of VI: The prevalence of VI in the present study is 23.5% (95% CI 20.5-26.9). The prevalence of different degrees of VI is shown in Table-I.

**Table-I:** Prevalence of different degrees of VI in relation to ages and genders.

Groups		Normal and Properly corrected	VI			Uni VI N (pre) (CI)	B/N N (pre) (CI)	All surveyed (100%)
			Mild VI N (pre) (CI)	Low vision N (pre) (CI)	Blindness N (pre) (CI)			
Gender	Male	208 (61%)	64 (18.7%) (14.8-23.4)	19 (5.5%) (3.4-8.7)	5 (1.4%) (0.5-3.5)	39 (11.4%) (8.3-15.4)	6 (1.7%) (0.7-3.9)	341
	Female	247 (68%)	51 (14%) (10.7-18)	20 (5.4%) (3.4-8.5)	7 (1.9%) (0.8-4)	32 (8.8%) (6-12.3)	7 (1.9%) (0.8-4)	364
Age	18- 30 Y	215 (73%)	31 (10.5%) (7.4-14.7)	7 (2.3%) (1-5)	2 (0.7%) (0.1-2.7)	34 (11.5%) (8.2-15.8)	6 (2%) (0.8-4.5)	295
	31-40 Y	108 (79%)	10 (7.3%) (3.7-13.4)	3 (2.2%) (0.5-6.7)	0	14 (10.2%) (5.9-16.8)	2 (1.5%) (0.25-5.7)	137
	41-50 Y	82 (75%)	13 (11.9%) (6.7-19.8)	3 (2.7%) (0.7-8.4)	1 (0.9%) (0.05-5.7)	7 (6.4%) (2.8-13.2)	2 (1.8%) (0.7-8.4)	109
	>50 Y	50 (30.5%)	61 (37.2%) (28.8-45)	26 (15.8%) (10-22.5)	9 (5.5%) (2.7-10.5)	16(9.7%) (5.8-15.6)	3 (1.8%) (0.1-4.8)	164
	Totals	455 (64.5%)	115 (16.3%) (13.7-19.3)	39 (5.5%) (4-7.5)	12 (1.7%) (0.9-3)	71 (10%) (8-12.6)	13 (1.8%) (1-3.2)	705

***Abbreviations:*** B/N; one eye blind and other eye normal, CI; confidence interval, N; number, Pre; Prevalence, Uni VI; unilateral visual impairment, VI; visual impairment.

There was no significant difference between male and female groups regarding their VI [p=0.9, χ^2^(df) = 1.572(6)]. However there was highly significant difference between different age groups in VI [p<0.0001, χ^2^(df) = 162.6(18)].

Causes of VI: Top five causes of VI with its different degrees are shown in Table II and III.

**Table-II:** Top Five causes of VI in relation to degree of VI.

Causes of VI 166 cases (100%)	Mild VI 115 cases (100%)	Low vision 39 cases (100%)	Blindness 12 cases (100%)
CAT 51 (30.7%)	CAT 33 (28.7%)	CAT 12 (30.7%)	CAT 6 (50%)
URE 41 (24.7%)	URE 31 (26.9%)	URE 6 (15.4%)	DR 2 (16.7%)
DR 22 (13.2%)	DR 15 (13%)	DR 5 (12.8%)	GL 2 (16.7%)
GL 14 (8.4%)	AMB 10 (8.6%)	GL 4 (10.2%)	CO 1 (8.3%)
AMB 13 (7.8%)	PCS 9 (7.8%)	KC 3(7.7%)	Trauma1 (8.3%)

***Abbreviations:*** AMB; amblyopia, CAT; Cataract, CO; corneal opacity, DR; diabetic retinopathy, GL; glaucoma, KC; keratoconus, PCS; post cataract surgery, URE; uncorrected refractive error.

**Table-III:** Top five causes of VIin relation to genders and ages.

Gender			Ages		

Males 88(100%)	Females 78(100%)	18 to 30 y 40(100%)	31-40 y 13(100%)	41-50 y 17(100%)	>50 96(100%)
CAT 26(29.5%)	CAT 25 (32%)	URE 27 (67.5%)	URE 7 (38.5%)	CAT 5 (29.4%)	CAT 41(42.7%)
URE 20 (22.7%)	URE 21 (27%)	AMB 6 (15%)	AMB 2 (15.4%)	DR 3 (17.6%)	DR 19(19.8%)
DR 9 (10.2%)	DR 13 (16.7%)	CAT 3 (7.5%)	CAT 2 (15.4%)	GL 3 (17.6%)	PCS 12 (12.5%)
GL 9 (10.2%)	PCS 9 (11.5%)	KC 3 (7.5%)	GL 1 (7.7%)	URE3 (17.6%)	CO 9 (9.4%)
AMB 7 (7.9%)	AMB 6 (7.7%)	Trauma 2 (5%)	Maculopathy 1 (7.7%)	Uveitis 3 (17.6%)	GL9 (9.4%)

***Abbreviations:*** AMB; amblyopia, CAT; Cataract, CO; corneal opacity, DR; diabetic retinopathy, GL; glaucoma, KC; keratoconus, PCS; post cataract surgery, URE; uncorrected refractive error.

Causes of unilateral VI: Forty four cases (62%) with unilateral VI were due to correctable causes while the remaining 27 cases (38%) were non-correctable. Refractive error was the commonest correctable cause of unilateral VI [25 cases (56.8%)], while amblyopia was the commonest non-correctable cause [12 cases (44.4%)].

Causes of unilateral blindness: In the studied population, 13 cases (1.8%) had shown unilateral blindness. Amblyopia was the commonest cause of unilateral blindness [6 cases (46%)].

## DISCUSSION

To the best of our knowledge it was for the first time that ophthalmic evaluation of the population in Arar city was performed to estimate the prevalence of VI and find out its leading causes.

Following WHO definition of VI, the present study data showed that the prevalence of VI was 23.5%, while the prevalence of blindness was 1.7%. These prevalences are the highest estimated prevalences in Saudi Arabia. Tabara and Ross-Degnan et al.12 stated that the prevalence of VI was 7.8%, while the prevalence of blindness 1.5%in the eastern province of Saudi Arabia. Al Faran et al.11 had reported 10.9% as prevalence of VI while 0.7% as prevalence for blindness in the South western region of Saudi Arabia. In a more recent study in Al-Jouf province (Alshaalan et al.9) had estimated 13.9% and 0.8% as prevalence of VI and blindness respectively. Furthermore our prevalence for blindness is higher than prevalence reported in nearby countries, like 0.6% in Lebanon^14^, 1.1% in Oman,^15^ 1.09% in Tehran.^16^ On the other hand, the prevalence of blindness reported in Pakistan (3.8% in rural and 2.5% in urban areas)^17^ is higher than our estimated prevalence. Unilateral VI was found in 10.07% of the studied population in the current study. The prevalence of unilateral VI was reported to be 7.3% in Australia.^18^

From the previous data we can conclude that comparisons must be done with great caution as a lot of factors may affect the final results of estimated prevalence. These include socioeconomic status, geographical location and efficacy of health care systems. In addition data are expected to be affected by examination methods and definitions of VI used in the different studies.

Regarding the causes of VI and blindness our data revealed that cataract is the leading cause of VI (30.7% of cases). This is in accordance with Al-Faran et al.^11^ in the south western region of Saudi Arabia. While in Al-Jouf study (Al-Shaaln et al.)^9^ refractive errors were reported as the leading cause and cataract as the second commonest cause. Internationally, prevalence of cataract was about 25% in Tehran,16 about one third of the studied population in England19 and 30% in Canada.^20^

Refractive errors (24.7% cases) were found to be the second main cause of VI in the current study. Uncorrected errors of refraction were shown as a leading cause of VI in many studies.^21^,^22^ Prevalence of RE was estimated to be 36% in Al-Jouf province, Saudi Arabia.^9^ Factors responsible for refractive errors remaining uncorrected include lack of awareness and recognition of the problem, non-availability of and/or inability to afford corrective measures and cultural disincentives to compliance.

The current study showed that diabetic retinopathy (DR) (13.2% cases) was the third main cause of VI. The higher incidence of DR as a cause of VI is expected as Saudi Arabia is the seventh leading country in incidence of diabetes. The prevalence of DR was estimated to be about 20% in the nearby Aljouf province.^9^

The present findings are important due to the following reasons; firstly, it confirms that the prevalence of VI and its main causes are different not only among countries, but also in the different provinces in the same country. Secondly, the study shows that we are in need for more periodic and obligatory ophthalmic evaluation as in the pre-occupational evaluation. Thirdly, the current data can help in better planning to decline rates of VI in the Northern Border Region.

## CONCLUSION

Visual impairment is a major global health problem. The results of the current study indicate that a large proportion of the visual impairment is treatable. Prevalence of VI is high in Arar city. Cataract, RE and DR are the major causes for VI in this part of the Kingdom. These findings are useful for better planning of eye care services in this region.
